# Body Mass Index and Associated Factors of School Absenteeism by School Feeding Program at Selected Primary Schools in Addis Ababa, Ethiopia: A Comparative Study

**DOI:** 10.1155/2021/6671468

**Published:** 2021-03-23

**Authors:** Solomon Muluken Ayehu, Addisu Tadesse Sahile

**Affiliations:** ^1^Department of Emergency, Menelik II Referral Hospital, Addis Ababa, Ethiopia; ^2^Department of Public Health, Unity University, Addis Ababa, Ethiopia

## Abstract

**Background:**

Quality of education plays a crucial role in the social, economic, and political development of a nation. Primary school is a vital stage in developing the personality and consciousness of school children.

**Objective:**

The study assessed the Body Mass Index and factors associated with School Absenteeism at selected primary schools in Addis Ababa, 2018.

**Methods:**

A comparative cross-sectional study was undertaken on 324 (162 each group) from selected primary schools of Addis Ababa from May 02 to July 30, 2018. All participants and their families provided written informed consent and assent. A systematic random sampling technique was used to select participants, where the list of students was once identified from the selected schools. An independent *t*-test was undertaken at *p* value <0.05 as the statistically significant level. And, binary logistics regression was used for the identification of factors statistically associated with school absenteeism, with its respective 95% confidence interval (CI) and *p* value of <0.05 significant level. *Findings*. There was a statistically significant difference between feeding and nonfeeding participants on average weight, school absenteeism, and BMI for age, at *p* < 0.05. Participants who enrolled in the school feeding program had a higher average weight than nonfeeding participants (*p* < 0.05). Participants from the feeding group had lower average school absenteeism than their counter participants (*p* < 0.05). The average BMI for age was significantly higher among feeding participants than nonfeeding participants (*p* < 0.05). The odds of having school absenteeism were 1.796 times higher among feeding participants than among nonfeeding participants (AOR: 1.796; 95CI:1.061–3.042, *p* < 0.05). The odds of sustaining absenteeism from the school were 2.257 times higher among feeding participants than among nonfeeding participants (AOR: 2.257; 95%CI: 1.291–3.948; *p* < 0.05).

**Conclusion:**

A higher number of school absenteeism, average weight, and BMI for age were observed in participants enrolled in the school feeding program than those who did not get enrolled in the feeding program. Large-scale studies were recommended to testify the impacts of school feeding on absenteeism.

## 1. Introduction

Quality of education plays a crucial role in the social, economic, and political development of a nation. Although progress was made, with a norm that education for all as part of Millennium Development Goals (MDG), about 58 million school-age children were out of school globally [1]. Assuring children acquiring basic knowledge and skills for their wellbeing could be the key responsibilities of all the governing bodies [2].

Malnutrition preceded by the inappropriate eating practice impacted adolescents' academic performance [3]. Though nutrition has a vital role in the development of humans including the brain throughout life [4], nutritional problems affect the ability of children to learn [5]. A balanced diet is more importantly advocated for productivity, cognitive wellbeing, endurance, and growth physically [6]. In sub-Saharan Africa, 25% of children sustained impaired mental development following malnutrition [7] and school children were at an increased risk of dropout and repetition of grades due to malnutrition [8].

Despite the higher burden of malnutrition among school children [9–12], where stunting affects up to 49% of children in Ethiopia and where underweight accounted of 59% [13], the coverage of school feeding program was low.

Although the school feeding program was introduced with the aim of increasing school attendance, enrollment, and retention, the coverage was low [14]. However, the impact of school feeding was not always positive on nutritional status; a corpus of evidence reported the presence of a positive link between school feeding and nutritional status [15–17]. Literature also reported the positive impact of the school feeding program on school attendance. Though school absenteeism was seen as a complex problem with multifactorial origin [18], the presence of a school feeding program reduces school absenteeism [15, 19–21].

To the best of the researcher's knowledge, the role of the school feeding programs on nutritional status and absenteeism was not well investigated in Addis Ababa. Therefore, this study assessed the BMI and school absenteeism within feeding versus nonfeeding groups in the study settings.

## 2. Methods

### 2.1. Participants and Study Design

The study received ethics approval from Sante Medical College, Research review and Ethics Committee, and was undertaken in selected primary schools of Addis Ababa from May 02 to July 30, 2018. All participants and their families provided written informed consent and assent. An institutional-based cross-sectional study was conducted at selected primary schools in Yeka subcity. The source population was primary school students in Yeka subcity. The study population was students at the selected primary schools. The selected schools were Kokebe Tsebah, Yewotatoch Genet, and Yeka Misrak Chora Primary Schools. A systematic random sampling technique was used to select participants, where the list of students was once identified. The sample size was determined with Epi-Data, based on double population formula presented as *n* = (*Zα*/2+*Zβ*)2*∗*(*p*1(1 − *p*1)+*pp*2(1 − *p*2))/(*p*1 − *p*2)2, where *Zα*/2 is the critical value of the Normal distribution at *α*/2 (for a confidence level of 95%, *α* is 0.05 and the critical value is 1.96), *Zβ* is the critical value of the normal distribution at *β* (for a power of 80%, *β* is 0.2 and the critical value is 0.84) and *p*1 and *p*2 are the expected sample proportions of the two groups. And finally, the sample size became 294, considering the 10% nonresponse rate; the final result was 324 for the two groups, with 162 for each group. Data were collected with a structured and pretested interviewer-administered questionnaire with an anthropometric measurement. A pretest was undertaken in Ewuket Fana Primary School on 5% of the sample size. The dependent variables were BMI and school absenteeism.

### 2.2. Operational Definitions

School absenteeism is any absence from school due to reasons other than the formal school closure days (due to either national holidays or religious days for which the school is closed). Interventional group: students who were included in the school feeding program from selected primary schools. Noninterventional group: students who were not part of the school feeding program but found in selected primary schools. WHZ: a child who had WHZ < −2 SD was categorized as low weight-for-height which implies undernutrition, and those who had WHZ > 2SD were regarded as overweight. WAZ**:** a child who had WAZ < −2 SD was categorized as low weight-for-age which implies undernutrition, and those who had WAZ > 2SD were regarded as high weight-for-age, overnutrition.

### 2.3. Statistical Analysis

After checked for completeness, the collected data were entered into Epi-Data 7 and then exported to SPSS 22 statistical software for further analysis. Descriptive statistics such as mean, percentages, standard deviation, and ranges were done. Comparisons on BMI and school absenteeism were performed with an independent Student's *t*-test, where a *p* value of less than 0.05 was a measure of statistically significant difference between the two groups. For the identification of factors statistically associated with school absenteeism, binary (bivariable and multivariable) logistics regression was applied, where fitness for the model was checked by the Hosmer–Lemeshow goodness of fit model. A 95% confidence interval and a *p* value of less than 0.05 were used as statistically significant levels.

## 3. Results

### 3.1. Sociodemographic Characteristics

A total of 324 participants aged 6–12 years old were enrolled from 3 selected primary schools. Of these, 162 were from the interventional group whereas 162 were from the control group and the response rate was 100%. Most (60%) of the feeding and more than half (54%) of the nonfeeding participants were male participants, while most (73%) of feeding and 78% of nonfeeding participants reported their father's presence within a family. The majority (92% and 91%) of the feeding and nonfeeding participants reported that they lived with their mothers, respectively. Physical measurements of participants depicted that 30% of feeding participants had a weight measurement of 30–34 KG, whereas 33% of the nonfeeding participants had a weight measurement of 25–29 kg (Table 1).

Most 70 % of the feeding and 77 % of the nonfeeding participants were absent from school due to illness, whereas only 14 % of feeding and 7 % of nonfeeding participants were absent from the school due to working for money and food (Table 2).

### 3.2. Body Mass Index Measurements

Most of the participants from both feeding and nonfeeding groups were found within the normal range of height-for-age measurements. Concerning BMI, most 98% of feeding and 90 % of nonfeeding participants were found within the normal range (between 2SD and 2 SD) (Table 3).

### 3.3. Comparison of Feeding versus Nonfeeding (*t*-Test Findings)

There was a statistically significant difference between feeding and nonfeeding groups concerning weight, the number of absent days from school, and BMI for age at *p* < 0.05.

Participants of the feeding groups had a higher average weight of 28.46 ± 6.554 as compared with the average weight of the nonfeeding groups, 27.00 + 4.81 (mean and standard deviation). Participants of feeding groups had a less (2.23 ± 2.420, mean, SD) average school absent days, as compared with the nonfeeding groups (3.96 ± 3.326, mean, SD) at *p* < 0.05 (Table 4).

### 3.4. Factors Associated with School Absenteeism

The odds of having had school absenteeism were 1.796 times higher among feeding participants than nonfeeding participants (AOR: 1.796; 95CI:1.061–3.042, *p* < 0.05). The odds of sustaining absenteeism from the school were 2.257 times higher among feeding participants compared with nonfeeding participants (AOR: 2.257; 95%CI: 1.291–3.948; *p* < 0.05). (Table 5).

## 4. Discussion

In this study, participants who enrolled in a school feeding program had a higher average weight than nonfeeding participants and were supported by the study in Vietnam. [22].

Though findings from this study revealed that there was no statistically significant difference on average last semester score between feeding and nonfeeding participants, a study in Argentina [23] stated that school feeding had a positive impact on the improvement of academic performance. A positive impact of the school feeding program on improvements in academic achievement was observed in different countries. Of these, it was observed in Nigeria [24], Kenya [25], Tanzania [26, 27], and Ethiopia [28] that the school feeding program had a positive impact on the academic performance of primary school students.

In this study, participants from the feeding group had lower average school absenteeism as compared to their counter nonfeeding participants. This indicated that, as students go through enrollment to the school feeding program, their tendency of school absenteeism tends to be lower and the same was reported by the studies in Ghana [29], Kenya [30, 31], and the southern part of Ethiopia [32].

Evidence from recommendation suggested that the school feeding program had a positive effect on the reduction of school absenteeism in primary school students. [33] On the contrary, a study in Ghana [34] stated that the school feeding program had no role in the reduction of school absenteeism. This variation might be due to differences in approach of delivery and population type.

In this study, the average BMI for age was significantly higher among feeding participants than nonfeeding participants and was supported by a study in Burkina Faso [35]. In this study, illness was one of the factors statistically associated with school absenteeism. Those students who sustained an illness had more risk of being absent from the school compared with their counterparts. Studies from Nigeria and Ghana also investigated that illness is one of the top factors affecting school absenteeism [36, 37].

School feeding programs came to be realized on the ground as part of the strategies to improve nutritional status, reduce absenteeism from school, and enhance academic performance. Studies in different parts of Ethiopia also reported the positive impact of the school feeding program on reducing the school absenteeism of primary school students [32, 38]. However, in this study, being enrolled in the feeding program had a negative impact on reducing school absenteeism. As a probable justification, this might be due to the fact that students from the feeding group might vary from those who did not enroll in the feeding program and hence they experienced more absenteeism than their counterparts.

## 5. Conclusions and Recommendations

Participants of the feeding groups had higher average weight than non-feeding groups, less average school absent days than their counterparts. The average BMI for age was also higher among the feeding group than those who did not enroll in the feeding program. The expansion of the program was recommended to be undertaken by the concerned stakeholders.

## 6. Limitations of the study

As this study compared nutritional status, academic performance, and absenteeism, it did not identify the factors behind those outcomes. And hence further studies were recommended.

## Figures and Tables

**Table 1:** Sociodemographic characteristics of participants at selected primary schools in Yeka subcity, Addis Ababa, Ethiopia, August 2018 (*n* = 162 for each).

Characteristics	Categories	Feeding		Nonfeeding	
		Number	%	Number	%
Sex	Male	96	60	88	54
	Female	66	40	74	46

Age in years	7	16	11	12	7
	8	14	9	16	10
	9	22	14	25	15
	10	25	14	23	14
	11	37	22	41	25
	12	48	30	45	28

Grade	Grade 1	27	17	27	17
	Grade 2	20	12	22	14
	Grade 3	24	15	26	16
	Grade 4	31	19	29	18
	Grade 5	30	19	24	15
	Grade 6	21	13	28	17
	Grade 7	9	5	6	3

Father presence	Yes	119	73	126	78
	No	43	27	36	22

Mother presence	Yes	149	92	147	91
	No	12	8	15	9

Religion	Orthodox	137	85	148	91
	Muslim	17	10	8	5
	Protestant	8	5	6	4

Birth order	First	63	39	62	38
	Middle	74	46	59	36
	Last	25	15	41	26

Weight of participants in kg	15–19	10	6.17	9	5.55
	20–24	42	25.92	42	25.92
	25–29	36	22.22	53	32.71
	30–34	49	30.24	47	29.01
	35 and above	25	15.43	11	6.79

Height in cm	<120	21	12.96	13	8.02
	120–129	32	19.75	23	14.19
	130–139	49	30.24	54	33.33
	140–149	44	27.16	53	32.71
	≥150	16	9.87	19	11.72

**Table 2:** School absenteeism and reasons for school absenteeism at selected primary schools in Yeka subcity, Addis Ababa, Ethiopia, August 2018 (*n* = 162).

Characteristics	Options	Feeding		Nonfeeding	
		Number	%	Number	%
School absenteeism due to illness	Yes	113	70	125	77
	No	49	30	37	23

School absenteeism due to working for money and food	Yes	23	14	11	7
	No	139	86	151	93

School absenteeism due to helping family by work	Yes	15	9	8	5
	No	147	91	154	95

School absenteeism due to hunger	Yes	5	3	2	1
	No	157	97	160	99

School absenteeism due to lack of interest to go to school	Yes	6	4	1	1
	No	156	96	161	99

School absenteeism due to other reasons	Yes	10	6	6	4
	No	152	94	156	96

**Table 3:** Nutritional status of participants at selected primary schools in Yeka subcity, Addis Ababa, Ethiopia, August 2018 (*n* = 162 for each).

Characteristics		Feeding status			
		Feeding		Nonfeeding	
		Number	%	Number	%
Height for age	<−2 SD	3	1.85	1	0.617
	−2 to 2 SD	154	95.06	154	95.06
	>2 SD	5	3.10	7	4.32

BMI for age	<−2 SD	0	0	11	6.79
	−2 to 2 SD	159	98.1	146	90.12
	>2 SD	3	1.85	5	3.10

**Table 4:** Nutritional status and school absenteeism of participants at selected primary schools, Addis Ababa, Ethiopia, August 2018 (*n* = 162 for each).

Characteristics		Feeding status	
		Feeding	Nonfeeding
		(*n* = 162)	(*n* = 162)
Weight	Mean ± SD	28.46 ± 6.554	27.00 ± 4.81^*∗*^
Height	Mean ± SD	1.35 ± 0.11	1.37 ± 0.11
BMI	Mean ± SD	15.55 ± 2.31	14.39 ± 2.07^*∗*^
Number of absent days from school	Mean ± SD	2.23 ± 2.420	3.96 ± 3.326^*∗*^
Last semester average mark	Mean ± SD	73.8746 ± 11.78	73.53 ± 12.61
Height for age	*Z* (mean ± SD)	2.01 ± 0.22	2.03 ± 0.22
BMI for age	*Z* (mean ± SD)	2.02 ± 0.13	1.96 ± 0.31^*∗*^

**Table 5:** Factors associated with school absenteeism at selected primary schools in Addis Ababa, Ethiopia, May 2018.

Characteristics	Categories	School absenteeism		COR (95%CI)	AOR (95%CI)
		Absent	Not absent		
Feeding status	Feeding	129	33	1	1
	Nonfeeding	114	48	1.646 (0.989–2.740)	1.796 (1.061–3.042)^*∗*^

Gender	Male	136	48	1	1
	Female	107	33	0.874 (0.524–1.456)	0.833 (0.481–1.442)

Grade of students	1–2	69	27	1	1
	3–4	86	24	0.713 (0.378–1.345)	0.743 (0.387–1.425)
	>5	88	30	0.871 (0.474–1.600)	0.757 (0.393–1.458)

Mother presence	Yes	223	74	1	1
	No	20	7	1.055 (0.429–2.594)	0.949 (0.373–2.417)

Birth order	First	92	33	1	1
	Middle	101	32	0.883 (0.503–1.550)	0.936 (0.524–1.672)
	Last	50	16	0.892 (0.448–1.777)	0.921 (0.454–1.869)

Illness	Yes	188	50	1	1
	No	55	31	2.119 (1.236–3.635)^*∗*^	2.257 (1.291–3.948)^*∗*^

^*∗*^ *p* < 0.05; ^*∗∗*^*p* < 0.001, statistically significant level.

## Data Availability

All data are already included in the manuscript.

## Abbreviations

BMI: Body Mass Index
FAO: Food and Agriculture Organization
HAZ: Height-for-age Z-score
MOE: Minster of Education
MOH: Minister of Health
MUAC: Midupper arm circumference
SFP: School feeding program
SPSS: Statistical package for social sciences
WAZ: Weight-for-age *Z*-score
WHZ: Weight-for-height *Z*-score.
